# Effect on 30-day mortality and duration of hospitalization of empirical antibiotic therapy in CRGNB-infected pneumonia

**DOI:** 10.1186/s12941-021-00421-2

**Published:** 2021-03-07

**Authors:** Rongrong Li, Hao Tang, Huaming Xu, Kunwei Cui, Shujin Li, Jilu Shen

**Affiliations:** 1 Department of Clinical Laboratory, The Second People’s Hospital of Hefei City, Hefei, Anhui Province China; 2 Fourth Affiliated Hospital of Anhui Medical University Laboratory, Hefei, 230012 China; 3 First Affiliated Hospital of Anhui Medical University Laboratory, Hefei, China

**Keywords:** Reasonable empirical antibiotics treatment, Bacterial pneumonia, Carbapenemase-producing, Gram-negative bacteria, Mortality

## Abstract

**Background:**

The objective of this study was to investigate whether unreasonable empirical antibiotic treatment (UEAT) had an impact on 30-day mortality and duration of hospitalization in bacterial pneumonia caused by carbapenem-resistant gram-negative bacteria (CRGNB).

**Methods:**

This was a retrospective cohort study involving CRGNB-infected pneumonia. All CRGNB-infected pneumonia patients received empirical and targeted antibiotic treatment (TAT), and they were divided into reasonable empirical antibiotic treatment (REAT) and UEAT according to whether the empirical antibiotic treatment (EAT) was reasonable. The data of the two groups were compared to analyze their influence on the 30-day mortality and hospitalization time in CRGNB-infected pneumonia patients. Moreover, we also considered other variables that might be relevant and conducted multivariable regression analysis of 30-day mortality and duration of hospitalization in CRGNB-infected pneumonia patients.

**Results:**

The study collected 310 CRGNB-infected pneumonia patients, the most common bacterium is *Acinetobacter baumannii* (211/310 [68%]), the rest were *Klebsiella pneumoniae* (46/310 [15%]), *Pseudomonas aeruginosa* and others (53/310 [17%]). Among them, 76/310 (24.5%) patients received REAT. In the analysis of risk factors, dementia, consciousness were risk factors of 30-day mortality, pulmonary disease, hemodynamic support at culture taken day and recent surgery were risk factors for longer hospital stay. The analysis of 30-day mortality showed that UEAT was not associated with 30-day mortality for the 30-day mortality of REAT and UEAT were 9 of 76 (11.84%) and 36 of 234 (15.38%) (*P* = 0.447), respectively. Meanwhile, there was difference between REAT and UEAT (*P* = 0.023) in the analysis of EAT on hospitalization time in CRGNB-infected pneumonia patients.

**Conclusions:**

UEAT was not associated with 30-day mortality while was related to duration of hospitalization in CRGNB-infected pneumonia patients, in which *Acinetobacter baumanniii* accouned for the majority.

## Introduction

EAT was carried out before the identification of bacteria and the determination of drug susceptibility. EAT for infections might be reasonable or unreasonable, that was, in vitro sensitivity with subsequently isolated pathogens, whether sensibility matched or not. For patients with severe infection, most clinicians tended to start EAT in the early stage of infection and the treatment scheme was often combined therapy [1]. At present, EAT has existed in the treatment of various infections, including bloodstream infections, urinary tract infections, pulmonary infections and so on [2–9], but the efficacy of EAT was not entirely certain. The excessive or unreasonable use of antibiotics were related to the increase of bacterial resistance, side effects and treatment costs. These problems were related to the treatment of infected patients by clinicians [10].

Patients admitted to ICU were prone to hospital-acquired pneumonia because of many underlying diseases, poor surrounding environment and low autoimmunity [11]. For some patients who needed catheter insertion, such as nasal catheter, mask, tracheal intubation and so on, bacteria easily entered the body through the catheter cavity and lead to catheter-related infection finally. The untimely treatment of such infection often resulted in death [12]. Based on this background, we studied the effects of EAT on 30-day mortality and hospital length of stay.

## Methods

### Study design

This was a retrospective cohort study involving CRGNB-infected pneumonia. All patients received EAT and TAT, and they were divided into REAT and UEAT according to whether EAT was reasonable. The data of the two groups were compared to analyze their influence on the 30-day mortality and hospitalization time in CRGNB-infected pneumonia patients. Cases were collected from January 2014 to March 2019 at Department of Laboratory Medicine, The First Affiliated Hospital of Anhui Medical University, a 2825-bed, tertiary teaching hospital in China. The first positive culture of each patient was taken as one sample in this study. The study was approved by the ethics committee of participating hospital.

### Participants

The study collected adult inpatients ≥ 18 years with hospital-acquired bacterial pneumonia or ventilator-associated bacterial pneumonia caused by CRGNB: *Acinetobacter baumannii, Pseudomonas aeruginosa,* or any *Enterobacteriaceae*. All inpatients received EAT and TAT.

### Definitions

REAT was defined as antibiotic therapy used within 48 h of bacterial culture and bacteria were sensitive to the antibiotics used in vitro drug sensitivity tests. Active antibiotic therapy sensitive to the corresponding bacteria after 48 h of culture was defined as TAT. 30-day mortality was defined as mortality at 30 days after the first positive culture isolate was taken from the CRGNB-infected patients. Hospitalization time was the number of days from the onset of infection to the discharge or death of patients with hospital-acquired infections. There were two clinical criteria for the diagnosis of bacterial pneumonia. First, there were obvious symptoms of cough and sputum, even fever, chest pain and so on. Second, chest X-ray or chest CT showed obvious patchy shadows in the lungs.

### Variables

The exposure variable was EAT and the outcome was 30-day mortality and hospital stay. Data were ascertained from patient written and electronic records and microbiology laboratory records. Besides, we considered other variables that might affect 30-day mortality and hospital stay in CRGNB-infected patients including patient demographics, basic disease, laboratory tests, unconsciousness, hemodynamic support, catheters, bacterial types, recent surgery and so on.

### Microbiology methods

Isolates were identified by MALDI-TOF MS systems (Bio Mérieux, Marcy l' Etoile, France). Antibiotic susceptibilities testing was performed by Clinical and Laboratory Standards Institute (CLSI) guidelines 2018. The broth dilution method was used to analyze the sensitivity of *Acinetobacter baumannii* and *Enterobacteriaceae* to tigecycline according to the interpretation standard established by FDA, When the minimum inhibitory concentration was less than or equal to 2 μg / ml, 4 μg / ml and more than 8 μg / ml, it was sensitive, intermediate and resistant separately.

### Statistical analysis

The study categorized the collected cases and compared data on survivors and deaths, as well as hospitalization time less than or equal to 30 days and more than 30 days. Proportions were compared using chi-square test, continuous variables using t-test or Mann–Whitney U test according to their distribution, the results were expressed as mean ± standard deviation and median (interquartile range) respectively. Risk factors for 30-day mortality or duration of hospitalization found significant on univariate analysis were entered into a multivariable logistic regression. Predictive performance of the model was assessed using Odds ratios (ORs) with 95% confidence intervals (CIs). Analyses were conducted using SPSS version 23.0 software.

## Results

A total of 310 eligible cases on CRGNB-infected pneumonia patients were collected, of which 76 (24.5%) were given REAT and the remaining 234 (75.5%) were UEAT. There were 105 (33.9%) female cases. The mean average age was 59.54 ± 16.59 years. Among them, *Acinetobacter baumannii, Klebsiella pneumoniae, Pseudomonas aeruginosa* and others were isolated from 211, 46 and 53 patients respectively, the cases collected were mainly concentrated in emergency department (183/310). The drug resistance of all CRGNB-infected pneumonia cases were shown in Table 1, Drug sensitivity showed that *Acinetobacter baumannii* and *Klebsiella pneumoniae* had high resistance to most antibiotics, and *Pseudomonas aeruginosa* present different degrees of resistance to each antibiotic. Similarly, all three bacteria showed low resistance to tigecycline, minocycline and polymyxin (< 8%).

**Table 1:** The drug resistance of all bacteria involved in the study

Antibiotics	Drug resistance rate		
	*Acinetobacter baumannii*	*Klebsiella pneumoniae* (%)	*Pseudomonas aeruginosa*
Ampicillin	/	100.00	/
Ampicillin/sulbactam	94.50%	100.00	/
Nitrofurantoin	/	96.55	/
Compound sulfamethoxazole	72.86%	47.50	/
Ciprofloxacin	91.90%	73.91	50.00%
Piperacillin/tazobactam	88.68%	67.39	39.13%
Gentamicin	75.24%	67.39	40.82%
Cefepime	94.29%	84.78	42.31%
Ceftriaxone	95.24%	97.50	/
Ceftazidime	94.76%	89.13	55.10%
Cefotetan	/	100.00	/
Cefoxitin	/	100.00	/
Tobramycin	70.81%	65.22	43.14%
Cefuroxime	/	100.00	/
Tigecycline	2.99%	3.85	/
Cefoperazone/sulbactam	/	73.33	/
Levofloxacin	48.80%	71.74	38.46%
Cefotaxime	95.83%	96.15	/
Minocycline	7.88%	0.00	/
Cefmetazole	/	75.00	/
Piperacillin	97.67%	76.92	43.48%
Cefazolin	/	100.00	/
Amikacin	57.67%	63.04	22.00%
Polymyxin	0.60%	0.00	3.33%
Aztreonam	/	97.67	75.00%

### Factors associated with duration of hospitalization

In this study, we listed risk factors that may be associated with 30-day mortality and duration of hospitalization, which were shown in Table 2 and Table 5. As shown in Table 2. For the analysis of hospitalization time, we categorized hospitalization time according to whether it was longer than 30 days and compared the two categories. Firstly, in univariate analysis, risk factors for hospitalization longer than 30 days were age, dementia, pulmonary disease, hemodynamic support at culture taken day, arterial line, central line, acquisition in ICU and recent surgery. Secondly, the result of multivariate regression analysis (Table 3) showed that pulmonary disease, hemodynamic support at culture taken day and recent surgery were risk factors for longer hospital stay. Finally, we divided EAT into REAT and UEAT to analyze the effect of EAT on hospitalization time (Table 4). The result showed that there was significant difference in length of stay between REAT and UEAT (P = 0.023).

**Table 2:** Factors associated with duration of hospitalization

Factors	≤ 30 days (n = 146)	> 30 days (n = 164)	P
Age	62.09 ± 17.88	57.28 ± 15.05	0.011
Gender			0.556
Male	99 (67.81%)	106 (64.63%)	
Female	47 (32.19%)	58 (35.37%)	
BMI (Kg/m^2^)	21.16 ± 4.31 (42)	22.61 ± 3.72 (83)	0.054
Congestive heart failure	31 (21.23%)	37 (22.56%)	0.778
Dementia	6 (4.11%)	1 (0.61%)	0.038
Pulmonary disease	21 (14.38%)	3 (1.83%)	0.000
Active malignancy			0.499
None	127 (86.97%)	135 (82.32%)	
Solid	15 (10.27%)	24 (14.63%)	
Hematological	4 (2.74%)	5 (3.05%)	
Liver disease			0.907
None	137 (93.84%)	152 (92.68%)	
Mild	8 (5.48%)	11 (6.71%)	
Severe	1 (0.68%)	1 (0.61%)	
Diabetes mellitus with end-organ damage	1 (0.68%)	2 (1.22%)	0.631
Renal disease	29 (19.86%)	38 (23.17%)	0.480
Total Charlson score	2 (1–3)	2 (1–3)	0.033
Creatinine (mg/dL)^a^	66.5 (48–106.55)	65.5 (47.1–139.4)	0.695
Albumin (g/dL) ^a^	33.54 ± 6.09	34.01 ± 6.15	0.502
WBC (× 10^9^/L) ^a^	11.26 ± 4.99	11.06 ± 5.2	0.584
Systolic blood pressure (mm Hg)^a^	126.01 ± 18.62	122.46 ± 17.4	0.084
Hemodynamic support^a^	57 (39.04%)	115 (70.12%)	0.000
Invasive ventilator support^a^	83 (56.85%)	105 (64.02%)	0.197
Normal consciousness	68 (46.58%)	82 (50%)	0.547
Arterial line	25 (17.12%)	47 (28.66%)	0.016
Urine catheter	106 (72.60%)	132 (80.49%)	0.101
Central line	57 (39.04%)	88 (53.66%)	0.010
Nasogastric tube	113 (77.40%)	131 (79.88%)	0.594
Acquisition in ICU	69 (47.26%)	96 (58.54%)	0.047
Recent surgery	43 (29.45%)	89 (54.27%)	0.000
Type of bacteria			0.623
*Acinetobacter*	101 (69.18%)	128 (78.05%)	
*Enterobacteriaceae (Klebsiella)*	16 (10.96%)	19 (11.58%)	
*Pseudomonas*/other	29 (19.86%)	17 (10.37%)	
EAT			0.266
REAT	40 (27.40%)	36 (21.95%)	
UEAT	106 (72.60%)	128 (78.05%)	
Covering empirical therapy by time			0.681
Same day as culture	32 (21.92%)	26 (15.85%)	
Day + 1	5 (3.42%)	5 (3.05%)	
Day + 2	3 (2.05%)	5 (3.05%)	

Data are presented as No. (%) unless otherwise indicated

^a^at culture taken day,

*BMI* body mass index, *WBC* white blood cell, *EAT* empirical antibiotic treatment, *REAT* reasonable empirical antibiotic treatment, *UEAT* unreasonable empirical antibiotic treatment, *ICU* intensive care unit

**Table 3:** Risk factors for duration of hospitalization, multivariate analysis

Factors	OR	95%CI		P
		Lower	Upper	
Age	0.995	0.98	1.01	0.511
Dementia	7.686	0.816	72.437	0.075
Pulmonary disease	5.049	1.395	18.274	0.014
Hemodynamic support at culture taken day	0.389	0.219	0.691	0.001
Arterial line	0.784	0.388	1.585	0.498
Central line	0.844	0.48	1.484	0.555
Acquisition in ICU	0.948	0.537	1.674	0.855
Recent surgery	0.421	0.25	0.708	0.001

**Table 4:** Comparison of hospitalization time between REAT and UEAT

Factor	REAT (n = 76)	UEAT (n = 234)	P
Duration of hospitalization	31.68 ± 11.56	34 (21–49.25)	0.023

### Risk factors for 30-day mortality

The total 30-day mortality was 16.98% (45/265), 9 out of 76 patients with REAT (11.84%) died, compared with 36 (15.38%) of 234 patients with UEAT (P = 0.447). In univariate analysis (Table 5). Congestive heart failure, dementia, unconsciousness, and recent surgery were risk factors for 30-day mortality, while other factors had no significant effect on the survival of patients. In addition, we classified EAT according to the time, and there was no significant effect on the survival of patients who received EAT at different time (P = 0.876). Moreover, EAT and significant risk factors (P < 0.05, Table 5) of 30-day mortality were analyzed by multivariable regression analysis. As shown in Table 6, after controlling covariates, UEAT was not a risk factor for 30-day mortality (OR 0.876, CI 95% 0.377–2.040, P 0.760).

**Table 5:** Risk factors for 30-day mortality

Factors	Alive	Dead	P
	(n = 265)	(n = 45)	
Age, mean ± SD	59.19 ± 16.24	61.62 ± 18.57	0.365
Gender			0.075
Male	170 (64.2)	35 (77.8)	
Female	95 (35.8)	10 (22.2)	
BMI, kg/m^2^	22.17 ± 4.98 (16)	22.12 ± 3.85 (109)	0.964
Congestive heart failure	53 (20.0)	15 (33.3)	0.046
Dementia	3 (1.1)	4 (8.9)	0.001
Pulmonary disease	20 (7.5)	4 (8.9)	0.756
Active malignancy			0.072
None	220 (83.0)	42 (93.3)	
Solid	36 (13.6)	3 (6.7)	
Hematological	9 (3.4)	0	
Liver disease			0.965
None	247 (93.2)	42 (93.3)	
Mild	16 (6.0)	3 (6.7)	
Severe	2 (0.8)	0	
Diabetes mellitus with end-organ damage	2 (0.8)	1 (2.2)	0.353
Renal disease	54 (20.4)	13 (28.9)	0.200
Total Charlson score	2 (0–10)	3 (0–8)	0.186
Creatinine (mg/dL)^a^	65.5 (21–926.8)	70.55 (18.6–463.3)	0.460
Albumin (g/dL)^a^	33.9 (3.9–62.8)	32.65 (16.5–45.9)	0.129
WBC (× 10^9^/L)^a^	11.17 ± 5.20	11.07 ± 4.60	0.906
Systolic blood pressure (mm Hg)^a^	124.09 ± 18.39	122.94 ± 16.15	0.520
Hemodynamic support^a^	151 (57.0)	21 (46.7)	0.208
Invasive ventilator support^a^	163 (61.5)	25 (55.6)	0.450
Normal consciousness	137 (51.7)	13 (28.9)	0.045
Arterial line	58 (21.9)	14 (31.1)	0.302
Urine catheter	202 (76.2)	36 (80.0)	0.580
Central line	122 (46.0)	23 (51.1)	0.529
Nasogastric tube	209 (78.9)	35 (77.8)	0.869
Acquisition in ICU	140 (52.8)	25 (55.6)	0.735
Recent surgery	119 (44.9)	13 (28.9)	0.045
Type of bacteria			0.992
*Acinetobacter*	196 (74.0)	33 (73.3)	
*Enterobacteriaceae (Klebsiella)*	29 (10.9)	6 (13.3)	
*Pseudomonas*/other	40 (15.1)	6 (13.3)	
EAT			0.447
REAT	67 (25.3)	9 (20.0)	
UEAT	198 (74.7)	36 (80.0)	
REAT by time			0.876
Same day as culture	48 (18.1)	7 (15.6)	
Day + 1	9 (3.4)	1 (2.2)	
Day + 2	7 (2.6)	1 (2.2)	

Data are presented as No. (%) unless otherwise indicated

^a^at culture taken day,

*BMI* body mass index, *WBC* white blood cell, *EAT* empirical antibiotic treatment, *REAT* reasonable empirical antibiotic treatment, *UEAT* unreasonable empirical antibiotic treatment, *ICU* intensive care unit

**Table 6:** Risk factors for 30-day mortality, multivariate analysis

Factor	OR	95%CI		P
		Lower	Upper	
Congestive heart failure	0.553	0.266	1.148	0.112
Dementia	0.125	0.025	0.629	0.012
Normal consciousness	2.538	1.236	5.213	0.011
Recent surgery	1.991	0.972	4.077	0.06
REAT	0.876	0.377	2.04	0.76

*CI* confdence interval, *OR* odds ratio, *REAT* reasonable empirical antibiotic treatment

## Discussion

In this work, we found that whether in univariate or multivariate analysis, the final results were consistent and stable, that was, UEAT did not increase the 30-day mortality, while it increased length of hospital stay of CRGNB-infected hospital-acquired pneumonia. Above data showed that UEAT prolonged the length of stay for about 2.5 days, the antibiotic regimens used in this study almost were combined therapy. This result only concerned patients with CRGNB-infected hospital-acquired pneumonia, and the main bacteria were *Acinetobacter baumannii* (68%). The same results had been found in previous studies [13–15]. However, other studies have found that UEAT could increase the 30-day mortality [16–20]_,_ which was different from our conclusion. This might be due to the high drug resistance rate of CRGNB which leading to serious illness and high mortality for CRGNB-infected pneumonia. This high mortality might real the role of REAT. But it did not mean that REAT has no effect on 30-day mortality. It might be that REAT can reduce 30-day mortality and hospitalization time of inpatients with mild and moderate diseases but has no obvious effect on severe patients [21]. Besides, in the study of Michek ST [22], it was found that UEAT did not increase mortality in patients with early-onset infection, but increased mortality in patients with late-onset infection. Therefore, there was no difference between UEAT and REAT in 30-day mortality in this article. Furthermore, other possible reasons for this result might lie in the differences of research type, age distribution, region, bacterial distribution, research object, sample size, variable control and so on.

In the analysis of hospital stay, pulmonary disease, hemodynamic support at culture taken day and recent surgery were risk factors for hospital stay, which suggested that inpatients with pulmonary disease, hemodynamic support at culture taken day and recent surgery would stay longer. UEAT could extend hospitalization samely. Therefore, clinicians should pay more attention to REAT to reduce the length of stay of patients. Moveover, the results of catheter insertion analysis showed that whether catheter, venous catheter, arterial catheter, ventilator or nasal catheter was not a risk factor for hospital stay. Which was also different from other literature [6]. The possible reason for this result was that the patient's serious condition did not reflect the influence of mechanical ventilation.

For the analysis of mortality, dementia, unconsciousness were risk factors for 30-day mortality, Clinicians could assess patients' survival status base on this result and thus provide corresponding survival support. In the analysis of REAT by time, we did not find any difference in mortality rate at different time in the early stage. However, literature report that the evaluation of 72-h empirical therapy was significantly correlated with the improvement of treatment rate, duration of antibiotic treatment and shortening of hospitalization time [6, 23]. The possible reason was that the number of our cases was too small to reflect the real results.

The advantages of our study were as follows: firstly, we analyzed a number of variables that might have impact on 30-day mortality and hospital stay, including patient demographic statistics, basic diseases, test results, infection and so on. Secondly, we controlled other variables and analyzed the influence of duration of hospitalization on 30-day mortality by multivariable regression analysis. Finally, we classified experiential therapy according to time and analyzed the data in many directions, which give direct and convenient results.

The shortcomings including the data were limited to the situation of hospital-acquired bacterial pneumonia in one hospital in recent years, there was no comprehensive evaluation of hospitals in different regions. Moreover, the study was limited to the hospital-acquired bacterial pneumonia by CRGNB. The situation of community-acquired pneumonia, bloodstream infection and other infections were not clear. In the selection of variables, only some of variables were selected. As we all know, in clinical death cases, any step and environment of patient's life could affect patient's survival, including economic status, medical environment, nursing situation, patient's psychological status and any other aspects. In the research of REAT according to time classification, our total data were less, which might not reflect the real situation, so further research was needed. And the results only assessed the impact of EAT on 30-day mortality and length of stay. Obviously, the causes of death of patients were not only infection and empirical therapy. In addition, there were literature focusing on the classification of therapeutic drugs of empirical antibiotics, including the effects of antibiotics alone and combinedly, which were not reflected in our study. The average age of the cases in the study was about 60 years old, which was limited to the comparison of the cases in the higher age group. *Acinetobacter baumannii* accounted for a large proportion of cases in the analysis, which only represented the results of this study. Furthermore, emergency department patients accounted for 55.2% (171/310) in this study.

In conclusion, for severe pneumonia with hospital-acquired CRGNB infection, UEAT did not increase the 30-day mortality, while increase the length of hospitalization. At the same time, the excessive or unreasonable use of antibiotics were related to the increase of bacterial resistance, side effects and treatment costs. Considering this, we recommend clinicians give REAT in the treatment of hospital-acquired CRGNB infection.

## Data Availability

The datasets generated and/or analysed during the current study are not publicly available due [REASON WHY DATA ARE NOT PUBLIC] but are available from the corresponding author on reasonable request.
